# Shifting and Recurring Partial Hemiplegia in a Patient Suffering from Bright’s Disease

**Published:** 1872-03-01

**Authors:** 


### SHIFTING AND RECURRING PARTIAL HEMIPLEGIA IN A PATIENT SUFFERING FROM BRIGHT'S DISEASE.

The following remarkable symptoms are supposed by Dr. Nicol, under whose care the patient was, and by his colleague, Dr. Bourne, to have been due to a shifting œdema about the pons Varolii.

J. W., aged thirty, suffering from Bright's disease, was admitted into the Bradford Infirmary, with extensive anasarca. His urine yielded more than half its bulk of albumen. After a course of hot-air baths, purgatives, and ferruginous tonics, the anasarca almost entirely disappeared, and the patient was enabled to get about. While in this condition, fifteen days after admission, he had, at midnight, an epileptiform seizure, which terminated in left facial and arm paralysis. He could not speak, but seemed to understand what was said to him. Mustard poultices were applied to the calves, and a catheter had to be passed.

On the following morning he was recovering slowly, and appeared to recollect all that had happened during the night. He spoke with difficulty. He was observed to be unable to close his eyes by a voluntary effort, although the usual winking took place. There were symptoms of lung congestion, for which poultices were applied. In the right axilla the temperature was 97·5°; in the left 97°. In the evening the patient's general condition had much improved. On the right side the temperature was 97·4°; on the left 99°. At ten o'clock in the evening another seizure occurred. This time the face was paralyzed on the right side; the eyes were drawn to the left; the power of speech was lost, he seemed unconscious, and yawned frequently. He passed urine in the usual quantity.

By ten o'clock on the following morning he was better, and pronounced simple words with difficulty. The face was not drawn, but the eyes were closed only with an effort. A grain of elaterium with four grains of calomel were placed on the tongue, and an enema of turpentine and castor oil was injected into the bowel. At ten in the evening a copious stool was passed, and in a quarter of an hour the power of speech returned. It was found that though in the morning he had been unable to swallow, and could not protrude the tongue beyond the teeth, he had taken his dinner well. The bowels were still further freely opened.

The next day, the fourth after the first seizure, urine was passed in usual quantities, and he seemed to be quite well. He was ordered five grains of tannin three times a day, with milk and sherry. He remembered those who had been present during his last attack (several persons being present), and affirmed that he had been conscious of all that had passed.

A third seizure followed at half-past one o'clock in the afternoon, after he had partaken fairly of dinner, and ended in right facial and arm paralysis, and partial loss of speech. Extensive dry cupping was practised for an hour; then an enema similar to the last was injected; a copious stool followed in rather more than an hour. The paralytic symptoms rapidly disappeared, and on the same day he was able to sit up in a chair.

On the following (fifth) day he was sitting up dressed; he spoke well, but the right arm was weak. He was ordered four ounces of sherry daily, and continued to take the tannin.

On the sixth day the urine was found to be acid, of a specific gravity of 1.010, and to contain pus-corpuscles to faint milkiness. It yielded rather less than one-half its bulk of albumen. Three days later the patient continued well, and the right arm was steadily gaining power.—*Lancet*, Sept. 16, 1861.—*Med. News*.

